# Clustering based on adherence data

**DOI:** 10.1186/1742-5573-8-3

**Published:** 2011-03-08

**Authors:** Sylvia Kiwuwa-Muyingo, Hannu Oja, Sarah A Walker, Pauliina Ilmonen, Jonathan Levin, Jim Todd

**Affiliations:** 1 School of Health Sciences, University of Tampere, Finland; 2 MRC Uganda Virus Research Institute, Entebbe, Uganda; 3 Medical Research Council Clinical Trials Unit, London, UK; 4 London School of Hygiene and Tropical Medicine, London, UK

## Abstract

Adherence to a medical treatment means the extent to which a patient follows the instructions or recommendations by health professionals. There are direct and indirect ways to measure adherence which have been used for clinical management and research. Typically adherence measures are monitored over a long follow-up or treatment period, and some measurements may be missing due to death or other reasons. A natural question then is how to describe adherence behavior over the whole period in a simple way. In the literature, measurements over a period are usually combined just by using averages like percentages of compliant days or percentages of doses taken. In the paper we adapt an approach where patient adherence measures are seen as a stochastic process. Repeated measures are then analyzed as a Markov chain with finite number of states rather than as independent and identically distributed observations, and the transition probabilities between the states are assumed to fully describe the behavior of a patient. The patients can then be clustered or classified using their estimated transition probabilities. These natural clusters can be used to describe the adherence of the patients, to find predictors for adherence, and to predict the future events. The new approach is illustrated and shown to be useful with a simple analysis of a data set from the DART (Development of AntiRetroviral Therapy in Africa) trial in Uganda and Zimbabwe.

## Introduction

Adherence is defined as the extent to which patients follow instructions for prescribed treatment necessary to achieve the full treatment benefits [1]. Treatment adherence is known to affect the outcome, but adherence behavior differs not only between patients, but also over time [2]. Also there is no standard measure of adherence, and different adherence measures (variables) are used in different settings and for different treatments [3]. For chronic diseases requiring continuous adherence to treatment a single measure is rarely useful, and one should use combined measures of adherence and consider both mean adherence and the variability in adherence over time [4].

To be clinically relevant, adherence measures should naturally be prominent predictors for future outcomes. With anti-retroviral therapy (ART) for HIV infection, some patients maintain viral suppression and achieve good outcomes with moderate levels of adherence to the newer drugs (boosted protease inhibitor and nonnucleoside reverse transcriptase inhibitors) [5]. However, good adherence is needed to minimise the potential for the emergence of resistance strains of the virus, and to support maximal survival benefit in the long-term [6].

In the analysis of adherence data we often use adherence as a predictor of future outcomes. In assessing the effect of an intervention, measures of adherence are also valuable in describing how well the intervention is received (which may change over time). One therefore wishes to understand and describe in a natural way the relationships

For example, in HIV infection several predictors of poor adherence to anti-HIV drugs can be found, including low socio-economic status of the patient [7], low education [8], regimen complexity [9], dosing frequency, cost of drugs and transport [10]. Non-adherence has also been associated with the drug regimen, personal factors, stigma, side effects, and travel away from home [11]. The impact of different patterns of adherence differs by drug class [5,12,13]. Various statistical methods have been used to predict adherence. Linear regression, logistic regression, or multinomial models have been used when adherence is expressed as a percentage, as a dichotomized variable (good vs poor adherence), or as a categorical or categorized ordinal variable (good, moderate, poor; 1,2,3+; etc.). Marginal models have been used for an analysis of repeated measures adherence data [14].

In the HIV studies, for example, adherence to ART is an important predictor of mortality, disease progression and virological failure [3,5,15]. Poor adherence to ART raises public health concerns of increased prevalence of disease, more potential for transmission of drug resistant virus to uninfected partners and minimizes the cost benefit of ART. However one difficulty with the analysis of adherence data is how to model the dynamic changes in adherence over time, and how to relate changes in adherence to patient characteristics, and to patient outcomes.

However since adherence is a dynamic and complex human behavior, the key is not so much the individual, observed values themselves, as whether we can characterize the underlying behavior of the patient outside the ART clinic from the observed pattern of reported adherence. In an alternative approach patient adherence measures are seen as a stochastic process, as described by Girard at al, Wong et al, and Sun et al [16], [17] and [18]. Stochastic models have the advantage of taking into account variability in adherence over time, being able to incorporate and distinguish missing data, and flexibility over the type of adherence measure used at each time point.

In our approach to develop new statistical tools to characterize and understand the adherence behavior of the HIV patients treated with ART, and to illustrate the use of these tool with real data. To do this the adherence measures at each time point are first categorized to a variable with finite number of values or "states". Repeated measures over time are then analyzed as a Markov chain of order 1, and the transition probabilities between the states are thought to describe the behavior of a patient. The patients are then clustered using their estimated transition probabilities between the various adherence states. These natural clusters can be used to describe variation in adherence, to find predictors for adherence, and to predict future disease progression or other outcomes. The new approach is illustrated with a simple analysis of a data set from the Development of Antiretroviral Therapy in Africa (DART) trial in Uganda and Zimbabwe (see http://www.ctu.mrc.ac.uk/dart). We compare the predictive powers of different models for mortality with adherence as a continuous and categorical explanatory variable under different Markov chain model assumptions. The comparisons are made using the ROC curves and areas under the ROC curves.

The paper is organized as follows. In Section 2 we describe the repeated adherence measurements on each individual as a Markov chain, and assume that the population consists of a finite number of clusters of patients with the same transition probabilities. In Section 3 the hierarchical clustering procedure based on the transition probabilities is described. Section 4 provides an example of DART trial data. We use three different models (repeated measurements are (i) independent and identically distributed, or distributed according to a (ii) homogeneous or (iii) non-homogeneous Markov chain model) to analyse the data, we describe and interpret the clusters and compare their ability to predict mortality. A discussion of the relative merits of this new approach is given in section 5.

### Adherence seen as a Markov chain

We assume that the adherence is measured by a discrete variable with finite number of possible values 1, ..., *S*. The values are here called *states*. For each individual the *states *are recorded at *T *time points 1, ..., *T *. The observed states are then denoted by *X*_1_, ..., *X_T _*, and the whole process can be seen as one classificatory variable with *S^T ^*classes or *profiles*. Note that if the adherence measurements are continuous or multivariate, they must first be categorized for the analysis. Note also that *missing data *at some time point can be treated as one of the states.

The adherence measurements over time points 1, ..., *T *are usually combined by averaging over the entire period to give the *estimated probabilities *(proportions) of being in each state,

where *I *(*X_t _*= *s*) = 1 if *X_t _*= *s *and zero otherwise. Note that, if *X*_1_, ..., *X_T _*are independent and identically distributed categorical random variables, this would be a sufficient way to describe the adherence behavior over the follow-up period. In this paper, we rather see the adherence as a process, as a (homogeneous) Markov chain with the *transition probabilities *between states

See Chapter 6 in [19], for example. Matrix

is then called the *transition matrix*. A natural estimate of *p_ij_*, if , is

A more complicated, and sometimes more realistic model to describe the adherence behavior is to use the *Markov chain of order 2 *with transition probabilities

Also, it is sometimes possible that the the Markov chain is *non-homogeneous *in the sense that the transition probabilities change at a time point t_1 _so that the transition probabilities are given by two *S *× *S *matrices, say,

### Clustering based on Markov chain approach

In the paper we assume that the adherence behavior of each individual is a Markov chain with unknown transition probabilities. We also assume that the population of the patients can be divided into subpopulations or clusters such that within a cluster the transition probabilities are the same. The cluster memberships can then be used as a categorical variable in further analysis. The unknown cluster memberships must naturally be estimated from the data.

The problem then is how to identify or estimate the clusters using the measurements

We explain the procedure in the case of the homogeneous Markov chain model. First we find the matrix *Q *= (*q_ij_*) with elements

and then vectorize *Q *to get a *S*^2^-variate observation vector

The vector *Z *is thus obtained by stacking the columns of *Q *on top of each other. Note that the estimates of the transition probabilities in *P *can be obtained by *Q *just by dividing each row of *Q *by its row sum. To avoid the possible divisions by zero we use *Q *instead of *P *in our analysis. The observed vectors *Z *are then used instead of the original *X*_1_, ..., *X*_*T *_to cluster the data.

In the following we use the *S*^2^-variate observations *Z*_1_, ..., *Z_N _*to cluster the *N *individuals in the data. (The vectors *Z_i_*, *i *= 1, ..., *N *, are in fact lying in the (*S*^2 ^- 1)-variate space as the sum of the components is one. We however prefer to use the whole vector *Z_i _*in our analysis instead of any choice of a subvector.) The marginal variables are often standardized for the cluster analysis but that is not natural here; the marginal variables are here probabilities and therefore already on the same underlying scale. We use the hierarchical clustering technique which starts with *N *clusters (individuals) and then iteratively joins the two most similar clusters until there is just a single cluster. This gives a *tree of clusters *with can be illustrated with a *dendrogram*; one can then cut the tree to have a suitable number of clusters. The clusters should not be too small and they should have relevant interpretations. Natural interpretations may be obtained via joint conditional transition probabilities in the cluster. A measure of dissimilarity or distance between classes is needed for the clustering procedure. Let two distinct index sets *I *and *J*, with *I*, *J *⊂ {1, ..., *N*}, give the indices corresponding to two clusters, and let *n_I _*and *n_J _*be the corresponding cluster sizes. The popular *Ward's minimum variance method *of linkage compares the between and within squared *Euclidean distances *with

where ,  and  are the sample mean vectors over the subsets with indices in *I *∪ *J *, *I *and *J*, respectively. See Chapter 7 in [20]. R software was used in the practical analysis of data.

If *X*_1_, ..., *X_T _*are identically and independently distributed then the clustering should be based on a S-vector *Z *= (*P*_1 _, ..., *P_S _*) only where , *s *= 1, ..., *S*. In case of the non-homogeneous Markov chain, one may consider two matrices of estimated probabilities, *Q*_1 _and *Q*_2_, which correspond to measurements at time points 1, ..., t_1 _and t_1 _+ 1, ..., T, respectively. The clustering algorithm is then based on the 2*S*^2^-vector *Z *= *vec*(Q_1_, Q_2_). The interpretations for the clusters can then made using two matrices of transition probabilities, *P*_1 _and *P*_2 _. In our application, the change point *t*_1 _is assumed to be fixed and known.

### An example: The DART trial in Uganda and Zimbabwe

#### The data and the problem

We illustrate the clustering procedure and its use with a cohort data set of 2960 participants in the DART trial in Uganda and Zimbabwe. The trial started in January 2003, and the patients were followed until the end of December 2008. Participants' adherence to the treatment was assessed by pill counts and a structured questionnaire administered at each scheduled 4-weekly clinic visit. Participants were asked questions on whether they had missed any dose in the last month, were late for the visit, had forgotten to take any dose at the weekend or missed any ART in the four days prior to the clinic visit. Drug possession ratio (DPR) previously defined as the days'supply of drugs delivered minus the days'supply of drugs returned divided by the number of days between clinic visits, assuming that ART was used continuously throughout the period between the clinic visits was obtained from clinic based pill counts [21]. Also missed clinic visits, death and loss to follow-up or withdrawals from the study were recorded.

The objective of this analysis was thus to find groups of DART patients with a similar adherence behavior (same proportions of being in each state, or same transition probabilities in homogeneous or non-homogeneous Markov chains) during the first year of the follow-up. We then considered whether the cluster membership could be explained by age or gender (chi-square tests for independence) and whether the risk of death during the second and third year of the follow-up was different in different clusters (chi-square goodness-of-fit tests, logistic analysis).

#### Adherence variables in this study

In this study we consider the adherence data collected during the first year of the follow-up period (*T *= 12 visits). Analysis was restricted to the *N *= 2960 patients who were alive at the end of the first year of the trial, as described in Muyingo et al [21]. We have previously shown that of all self reported measures, 'missed any dose in the last month' most strongly associated with Viral load [21]. The adherence patterns of patients alive at 12 months were later used to predict the mortality during the second and third year. Adherence data were missing at a visit either because the patient (i) totally missed a visit, or (ii) attended but did not complete the adherence questionnaire. For the illustration of our approach here we only use a simple binary variable 'missed any dose in the last month' with the third possibility of missing data for this question for any reason. We then use the Markov chain with *S *= 3 states,

Note that the adherence values (*X*_1_, ..., *X_T _*) may be seen as one classificatory variable with *S^T ^*= 3^12 ^= 531, 441 classes (profiles). Clustering is a way to reduce the number of classes in a rational way.

The rational and efficient use of the observed values *X*_1_, ..., *X_T _*in the clustering naturally depends on the true statistical model. In the following we consider and compare three different models, namely,

(M1) the i.i.d. model, that is, *X*_1_, ..., *X_T _*identically and independently distributed,

(M2) the homogeneous Markov chain model, and

(M3) the non-homogeneous Markov chain model.

In the model (M3) we assume that the change point is at six months time. Note that the models are nested so that (M1) ⇒ (M2) ⇒ (M3). Likelihood ratio tests can be used to discriminate between the models.

To illustrate the differences between the approaches, consider three individuals, *i*_1_, *i*_2_, and *i*_3_, with the following observed values of *X*_1_, ..., *X*_12_.

The profiles of the individuals look very different but, if we assume that the model (M1) is true and therefore use estimated probabilities of being in each state (sufficient statistic), the we get

and the three individuals are treated identically in the analysis ().

If one assumes that the second model (M2) is true then one should use the matrix *Q *= (*q_ij_*) with elements

(a sufficient statistic) to condense the data. For the three individuals we then get

and individuals *i*_2 _and *i*_3 _again get the same value *Z = vec*(*Q*). Finally, in the third model (M3), the three values of *Z = vec*(*Q*_1_, *Q*_2_) are different, namely,

Depending on the chosen model one then uses the adherence variable

in the analysis. The variable is then 3-, 9- or 18-variate, respectively.

#### Clustering based on the models (M1), (M2), and (M3)

We use hierarchical clustering as explained in Section. The clustering is first based on the 3-variate variable *Z *= *P *, and the number of clusters was chosen to be six. The probabilities of being in states 0,1, and 9 in each cluster are reported in Table 1. Cluster 1, for example, has the highest proportion for missing data but the proportion for good behavior is also high 83%. Cluster 5 is the poorest one as the proportion for good behavior is only 43%. The last cluster 6 consists of 891 optimally behaving patients.

**Table 1:** The probabilities of being in state 0, 1, and 9 in six clusters based the i.i.d. model (M1).

	State			
	0	1	9	Σ
Cluster 1 (n = 469)	.065	.829	.107	1.000
Cluster 2 (n = 426)	.280	.688	.032	1.000
Cluster 3 (n = 360)	.167	.833	.000	1.000
Cluster 4 (n = 618)	.083	.917	.000	1.000
Cluster 5 (n = 196)	.489	.426	.085	1.000
Cluster 6 (n = 891)	0	1	0	1.000

The clustering is next based on the 9-variate variable *Z *= *vec*(*Q*) and the homogeneous Markov chain model. For the comparison we use again six clusters here. The transition probabilities between states 0, 1, and 9 in six clusters are reported in Table 2. The probabilities in the table for cluster 2, for example, are read as follows. 52% of those patients who reported good adherence in the previous month achieved good adherence also in this month, 31% of those having missing data in previous month reported good adherence in this month, and so on.

**Table 2:** Conditional transition probabilities in six clusters based on homogenous Markov chain model (M2)

	**Cluster 1 (*n *= 301**)					Cluster 2 (*n *= 281)			
									
	0	1	9	Σ		0	1	9	Σ
0	0	1	0	1.000	0	0.425	0.523	0.052	1.000
1	0.008	0.950	0.042	1.000	1	0.436	0.516	0.049	1.000
9	0	1	0	1.000	9	0.235	0.307	0.458	1.000
	**Cluster 3 (*n *= 463)**					**Cluster 4 (*n *= 596)**			
									
	**0**	**1**	**9**	**Σ**		**0**	**1**	**9**	**Σ**
0	0.177	0.765	0.057	1.000	0	0.253	0.723	0.024	1.000
1	0.073	0.871	0.056	1.000	1	0.207	0.770	0.023	1.000
9	0.131	0.652	0.217	1.000	9	0.222	0.684	0.094	1.000

	**Cluster 5 (*n *= 469)**					**Cluster 6 (*n *= 850)**			
									
	**0**	**1**	**9**	**Σ**		**0**	**1**	**9**	**Σ**
0	0	1.000	0	1.000	0	.	.	.	.
1	0.091	0.909	0	1.000	1	0	1.000	0	1.000
9	.	.	.	.	9	.	.	.	.

Cluster 2 is clearly the poorest one, as the proportion maintaining good adherence from one month to the next is the lowest. Patients in cluster 6 behave in an optimal way.

Third, we also clustered the data using 18-variate variable *Z *= *vec*(*Q*_1_, *Q*_2_) based on the heterogeneous Markov chain model. The conditional transition probabilities were then allowed to be different over different periods (with a change point at six months). The estimated transition probabilities with six clusters are given in Table 3. The clusters can be roughly characterized in the following way.

**Table 3:** Conditional transition probabilities in six clusters based on heterogeneous Markov chain model (M3)

	Cluster 1 (*n *= 519)								
		Period 1					Period 2		
									
	0	1	9	Σ		0	1	9	Σ
0	0.027	0.960	0.133	1.000	0	0.095	0.866	0.039	1.000
1	0.034	0.953	0.013	1.000	1	0.115	0.824	0.061	1.000
9	0.117	0.860	0.023	1.000	9	0.051	0.800	0.149	1.000
	**Cluster 2 (*n *= 309)**								
		**Period 1**					**Period 2**		
									
	**0**	**1**	**9**	**Σ**		**0**	**1**	**9**	**Σ**
0	0.431	0.526	0.043	1.000	0	0.403	0.549	0.048	1.000
1	0.497	0.458	0.045	1.000	1	0.279	0.672	0.049	1.000
9	0.264	0.373	0.364	1.000	9	0.123	0.352	0.519	1.000

	**Cluster 3 (*n *= 408)**								
		**Period 1**					**Period 2**		
									
	**0**	**1**	**9**	**Σ**		**0**	**1**	**9**	**Σ**
0	0.275	0.684	0.041	1.000	0	0.000	1.000	0.000	1.000
1	0.246	0.690	0.063	1.000	1	0.015	0.978	0.007	1.000
9	0.226	0.598	0.177	1.000	9	0.067	0.933	0.000	1.000

	**Cluster 4 (*n *= 441)**								
		**Period 1**					**Period 2**		
									
	**0**	**1**	**9**	**Σ**		**0**	**1**	**9**	**Σ**
0	0.285	0.681	0.035	1.000	0	0.191	0.781	0.028	1.000
1	0.163	0.799	0.039	1.000	1	0.273	0.697	0.030	1.000
9	0.202	0.556	0.242	1.000	9	0.261	0.620	0.120	1.000

	**Cluster 5 (*n *= 433)**								
		**Period 1**					**Period 2**		
									
	**0**	**1**	**9**	**Σ**		**0**	**1**	**9**	**Σ**
0	0.000	1.000	0.000	1.000	0	.	.	.	.
1	0.118	0.853	0.029	1.000	1	.	1.000	.	1.000
9	0.000	1.000	0.000	1.000	9	.	.	.	.

	**Cluster 6 (*n *= 850)**								
		**Period 1**					**Period 2**		
									
	**0**	**1**	**9**	**Σ**		**0**	**1**	**9**	**Σ**
0	.	.	.	.	0	.	.	.	.
1	.	1.000	.	1.000	1	.	1.000	.	1.000
9	.	.	.	.	9	.	.	.	.

• Cluster 1: Good adherence - getting worse

• Cluster 2: Poor adherence with missing data - getting slightly better

• Cluster 3: First poor with some missing data - then very good

• Cluster 4: Poor with some missing data - no big changes

• Cluster 5: First good - then optimal

• Cluster 6: Optimal in both periods

Table 4 gives a cross-tabulation of cluster memberships in the three clustering based on models (M1), (M2), and (M3). One can see that the groups are genuinely different and, as seen from the description of clusters above, the groupings based on (M2) and (M3) describe the adherence behavior in more versatile ways.

**Table 4:** Contingency tables for cluster categories when clusters are based on (a) models (M1) and (M2), (b) models (M1) and (M3), and (c) (M2) and (M3).

	**(a) (M2)**						
		**1**	**2**	**3**	**4**	**5**	**6**
		
(M1)	1	146	1	250	72	0	0
	2	0	11	6	309	0	0
	3	0	0	172	188	0	0
	4	114	0	35	0	469	0
	5	0	169	0	27	0	0
	6	41	0	0	0	0	850
		
	**(b) (M3)**						
		**1**	**2**	**3**	**4**	**5**	**6**
		
	1	187	14	116	89	63	0
	2	5	127	82	212	0	0
	3	63	1	187	109	0	0
	4	223	0	19	6	370	0
	5	0	167	4	25	0	0
	6	41	0	0	0	0	850
		
	**(c) (M3)**						
		**1**	**2**	**3**	**4**	**5**	**6**
		
(M2)	1	124	0	0	0	177	0
	2	0	223	0	58	0	0
	3	158	2	252	51	0	0
	4	24	84	156	332	0	0
	5	213	0	0	0	256	0
	6	0	0	0	0	0	850

#### Adherence clusters, predictors and explanatory variables

As an illustration of the use of the clusters in a further analysis, we considered the relationship between age and sex and adherence which was categorized using clusters based on non-homogeneous Markov chain model (M3). We also considered how the risks of death in the second and third year of follow-up were associated with adherence behavior during the first year. We thus follow the scheme

The results in the analyses for clusters coming from heterogeneous Markov chain model (*Z *= vec(*Q*_1_, *Q*_2_)) are given in Tables 5 and 6. No difference was found between the proportions of women or between the age distributions. There were 100 deaths in the second and third year, individuals in cluster 2 were 2.72 (95% CI:1.42 to 5.18) times more likely to die and in cluster 4 were 2.08 (95% CI:1.42 to 5.18) times more likely to die as compared to Cluster 6 with optimal adherence. Individuals in Clusters 1 and 3 were 1.41 (95% CI:0.72 to 2.71) and 1.47 (95% CI:0.72 to 2.92) times more likely to die whilst in cluster 5, were 0.88 (95% CI:0.72 to 2.71) times likely to die compared to the optimal cluster 6. Adjusting for age and sex did not change the OR estimates. (Age and sex are not confounding factors in this analysis.) Again, R software was used in these analyses.

**Table 5:** Clusterwise mortality in the second and third year on ART, proportion of women and proportion of patients in three age groups.

				Age at ART initiation		
	n	deaths	women	18-35	35-45	45+
Cluster 1	(*n *= 519)	.033	.65	.41	.42	.17
Cluster 2	(*n *= 309)	.061	.62	.39	.42	.19
Cluster 3	(*n *= 408)	.034	.65	.41	.42	.16
Cluster 4	(*n *= 441)	.048	.65	.41	.43	.16
Cluster 5	(*n *= 433)	.020	.65	.37	.45	.15
Cluster 6	(*n *= 850)	.024	.65	.40	.46	.15

Six clusters are based on the heterogeneous Markov chain model.

**Table 6:** Estimated odds ratios (OR) with 95 percent confident intervals to compare the risk of deaths in different clusters, also adjusted for age and sex.

	OR	95% CI	Adjusted OR	95% CI
Cluster 1	1.4	(0.72, 2.71)	1.4	(0.71, 2.68)
Cluster 2	2.7	(1.42, 5.18)	2.7	(1.41, 5.17)
Cluster 3	1.5	(0.72, 2.93)	1.5	(0.71, 2.91)
Cluster 4	2.1	(1.11, 3.89)	2.1	(1.10, 3.87)
Cluster 5	0.9	(0.38, 1.90)	0.88	(0.38, 1.90)
Cluster 6	1		1	

Cluster 6 serves as a reference class. The clusters are based on the heterogeneous Markov chain model.

**Figure 1:**
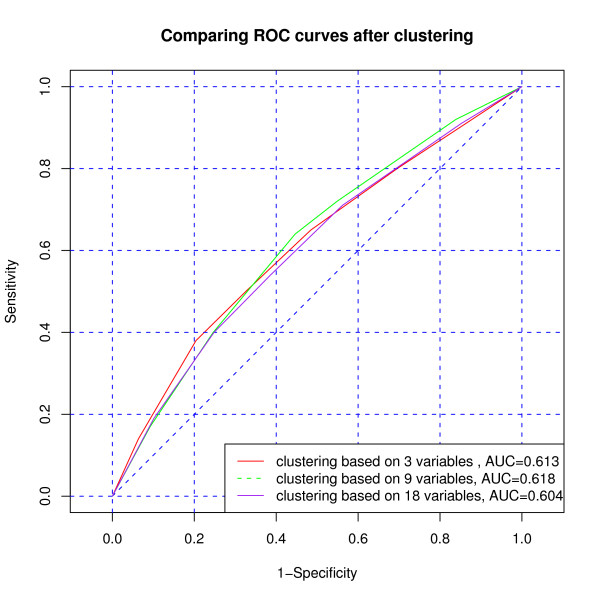
**ROC curves for predicting mortality using categorigal cluster variable based on model (M1) (3 variables), model (M2) (9 variables) and model (M3) (18 variables)**.

**Figure 2:**
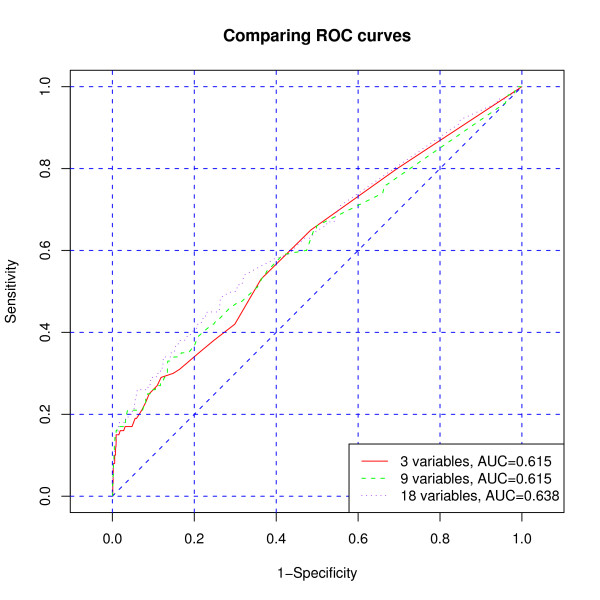
**ROC curves for predicting mortality using continuous *Z*-variable based on model (M1) (3-variate), model (M2) (9-variate) and model (M3) (18-variate)**.

Finally, we also compared the categorical cluster variables based on the three models (M1), (M2), and (M3) as predictors of mortality during the second and third year of ART. If linear predictor *β'Z_i _*with the rule *β'Z_i _*>*c *is used as a predictor for the death of individual *i*, then the receiver operating characteristic (ROC) curve is a graphical tool for illustrating the trade off between the false negative (sensitivity) and false positive rates (specificity) for all possible cut off points *c*, and the area under the ROC curve is a numerical measure of that. There are no big differences between the predictive powers of the three categorical cluster variables based on models (M1), (M2), and (M3) as seen in Figure 1. For the comparison, also the ROC curves from the conventional logistic regression with explaining variables *Z *= *P *, *Z *= *vec*(*Q*), and *Z *= *vec*(*Q*_1_, *Q*_2_) used as linear predictors in the conventional logistic regression model are given in Figure 2. (Of course the linearity assumption may not be realistic.) The predictive powers in these cases were again very similar. This may be due to the very short follow-up time of 12 months to assess adherence.

## Discussion

The main motivation of this paper was to develop and illustrate new statistical tools to characterize and understand the adherence behavior of the HIV patients treated with ART, and to illustrate these tools with data from the DART study. The Markov chain model is perhaps the simplest model for dependent categorical repeated measurements. In this work, estimated proportions and transition probabilities in a three-state Markov chain model were used to cluster the individuals into groups with different adherence patterns. Transition probabilities represent the dynamics of adherence to ART, and the non-homogeneous Markov chain model allows the patients to change their adherence behaviour over time. Relating patient characteristics to transition probabilities may enable a better understanding of adherence patterns compared to the traditional methods of just averaging the raw adherence data.

We illustrated the approach using one variable - "missed any dose of ART in the last month" - but the approach could be extended to a any number of adherence variables. Several variables can then be used together to construct the states needed for Markov chain model. Our approach can naturally be applied to different data sources (patients diaries, electronic event monitoring, drug possession ratio %), and to the adherence to several drugs simultaneously. Using different adherence variables (if not highly correlated) would produce different clusters with different predictive powers for mortality. The choice of adherence variables is therefore a crucial step, and depends on the data and application.

In our approach, the adherence observations *X*_1_, ..., *X_T _*at time points 1, ..., *T *, are seen as a realization of a random process. Data analysts often implicitly assume that the observed values *X*_1_, ..., *X_T _*are independent and identically distributed (iid). We think that such assumptions should be explicitly stated, and that it is unrealistic to assume that there is no dependence and no changes in distributions of *X_i_*. With our Markov chain model we have explicitly stated and assumed a certain simple dependency between the observations. In the paper, we compare three different models, namely the iid model (M1), the homogenous Markov chain model (M2), and the non-homogenous Markov chain model (M3). Note that both (M1) and (M2) assume that there is no change in the adherence behavior over the follow-up period. In model (M3) this is allowed. If we assume constant transition probabilities over time we may lose information and important aspects of the phenomenon. It also important to note that the three models are nested within each other, so that the regular likelihood ratio tests can be used to distinguish between the models; this will be studied in our future work. In the paper we are interested in modeling adherence behavior in general rather than in modelling the changes in adherence behavior as in Lazo et al [22]. However, our model (M3) is flexible enough for modeling the changes as well. In model (M3), the transition probabilities can naturally be made to depend on explaining (modifiable) factors; this is however beyond the scope of this paper.

In our earlier paper [21] we showed that although adherence looked very high overall in the first year in DART, this masked an inconsistent adherence behavior at the individual level. For the illustration and comparison of different adherence profiles we gave an example of three individuals with very different profiles ((9, 9, 9, 0, 0, 0, 0, 0, 1, 1, 1, 1), (9, 0, 1, 1, 1, 9, 1, 0, 0, 0, 0, 9), and (9, 1, 0, 0, 0, 9, 0, 0, 1, 1, 1, 9)) but similar overall adherence (measured as proportions). The differences between the individuals cannot be explained with model (M1) and not even with model (M2). Only model (M3) can analyse the differences between these three individuals. For the DART data set, we found six clusters based on models (M1), (M2), and (M3). The clusters were genuinely different with different interpretations. Also clusters with changing behavior could be found (which supports the use of model (M3)). Our findings suggest that different approaches may be potentially useful in practical data analysis, and that overall (mean) adherence may not be enough when dealing with ART adherence. We compare the predictive powers of the procedures based on models (M1), (M2), and (M3) for mortality with ROC curves. We could not find any big differences between the procedures, but this may just be due to the short period for the measurements of adherence. Whilst viral load is of major importance to participants, this was not done in real-time, so not available for all participants. Death was also relatively infrequent in the second and third year (proportion of deaths = 3%) and so the power to distinguish different effects of M1 from M2 or M3, M2 from M3 was low. However the fact that M2 and M3 classified people differently illustrates potential of our approach.

The DART trial has collected data on virological failures and immunological failures as well. Those outcome variables will be considered in future analyses looking at the predictive value of these adherence clusters. We did not find any significant association between the adherence groups and age or gender. It is possible, however, that other socio-demographic variables are associated with the adherence groups. This approach could then be used in the future to identify individuals at risk of poor adherence. It is of course important to validate this new approach against outcomes that are associated with adherence to ART. In this work, the clustering variable based on the non-homogeneous Markov model was seen to be associated with the risk of death in the second and third year of ART. Those reporting poor or less than adequate adherence had a significantly higher mortality in the second and third year than those that achieved optimal adherence, with the good and adequate users somewhere in-between. The worst cluster has the highest mortality risk.

Our analysis was restricted to those who survived the first year of the trial [21]. The majority of deaths in the first year occurred in the first 3 months (50%). The patients with early deaths did not have the opportunity to fully demonstrate their adherence behavior. We also reasoned that poor patient outcome associated with adherence would likely manifest later in the course of treatment. We considered mortality during the second or third year as the outcome that adherence might predict. The causal pathway is that poor adherence leads to viral replication in the presence of low levels of drug which leads to drug resistance which leads to viral rebound which leads to CD4 decline, and finally leads to morbidity/mortality. It is precisely this process which takes several months, and motivates our prediction model where adherence in the first year predicts mortality in the second or third year.

Because we have used a relatively short time period for the assessment of adherence, the vector *Z *(with dimensions 3, 9, and 18 in different models) that we use for clustering seems to have a higher dimension than the vector of original observations (with dimension 12). However, the original adherence measurements yield in fact 3^12 ^different profiles, and the idea here is to classify these profiles in a rational way. The transformed variables Z provide sufficient statistics in different models; if *Z *is known then *X*_1_, ..., *X_T _*does not carry any additional information on the model. Our clustering procedures were based on Euclidean distances and Ward's minimum variance method as they seemed to work well in our case. Alternative linkage methods and distance measures should be used to generate the clusters for the comparison. In determining the distances between the individuals we could for example assign different weights to different transition probabilities. This will be a part of our future work on the use of stochastic adherence models and their use to predict future events.

It is of course not always clear what population quantities we are estimating when we report the odds ratio estimates for mortality for the six clusters we have obtained. The underlying assumption is that the data set used in the analysis is a random sample from a population with six subpopulations having different adherence behavior, then the cluster memberships (with six clusters) estimate the unknown subpopulation memberships, and the odds ratios using cluster memberships estimate the unknown odds ratios for the difference in mortality in the subpopulations. Under the above strict assumptions one may hope that the estimates are consistent to population values. However, if one does not believe in this assumption, one can still consider the predictive power of the whole procedure (area under the ROC curve) and use that for meta-analysis.

There are several extensions and possibilities to develop and deepen the analysis of DART trial data: Another extension to this approach would be to use two or more measures of reported adherence for the states in the Markov chain model. Continuous measurements such as the drug possession ratio could be categorized and used in the Markov chain model. One could look at trends over time and/or over a longer period of 3 years. One could use more states such as lost in the follow-up. In our analysis the problem of drop-outs did not arise as all patients who died or were lost to follow-up in the first year of the trial had been excluded from the dataset [21]. Only 968(2.7%) clinic visits had missing data, 653 were due to forms not being completed by the adherence nurse and 315 were due to missed visits. These numbers were small and were not divided further in this application but could easily be divided if the analysis required it.

For example in clinical trials, non-response or drop-out are important outcomes in their own right and should be distinguished from incomplete forms or poor documentation. Another extension of the model used here would be the use of the Markov chain model of order 2. Statistical tools are needed for the model selection: Statistical tests and estimates for the change point in a non-homogeneous Markov chain model, and tests and estimates for the order of the model. We could easily build likelihood ratio tests for our nested parametric families of distributions. To show whether our Markov chain fits better than an independence model and more specifically test for homogeneity; if our non-homogeneous Markov chain of order 2 fits better than the homogeneous one.

Our aim in this paper was to develop and illustrate some new ideas on how to classify patients based on adherence data using a stochastic model and to illustrate how this analysis could be carried out on real data. Further detailed analyses will be undertaken using the full DART dataset, in which we will explore the extensions to the basic model, develop ways of testing different models and evaluate factors that influence the transition probabilities. We believe this may develop a new way of looking at adherence and in better analysing adherence data.

## Competing interests

The authors declare that they have no competing interests.

## Authors' contributions

All authors read and approved the manuscript prior to submission, and approved the final manuscript. All authors have agreed with revisions to the manuscript during the submission process. SK-M wrote the first draft of the manuscript. SK-M and PI did the statistical analyses of the data. SK-M and HO conceived the use of Markov chain models in the analysis of adherence data. SK-M, JL, JT and SW conceived the analysis of adherence data from the DART trial. SK-M and SW prepared the data from the DART trial for analysis.
